# Exosomes derived from cancerous and non-cancerous cells regulate the anti-tumor response in the tumor microenvironment

**DOI:** 10.18632/genesandcancer.172

**Published:** 2018-03

**Authors:** Susan Bae, Jeffrey Brumbaugh, Benjamin Bonavida

**Affiliations:** ^1^ Department of Oral Biology, UCLA School of Dentistry, University of California, Los Angeles, CA, USA; ^2^ Department of Microbiology, Immunology & Molecular Genetics, David Geffen School of Medicine, University of California, Los Angeles, CA, USA

**Keywords:** angiogenesis, exosomes, immunoregulation, immunotherapy, tumor-derived exosomes

## Abstract

The tumor microenvironment (TME) is a unique platform of cancer biology that considers the local cellular environment in which a tumor exists. Increasing evidence points to the TME as crucial for either promoting immune tumor rejection or protecting the tumor. The TME includes surrounding blood vessels, the extracellular matrix (ECM), a variety of immune and regulatory cells, and signaling factors. Exosomes have emerged to be molecular contributors in cancer biology, and to modulate and affect the constituents of the TME. Exosomes are small (40-150 nm) membrane vesicles that are derived from an endocytic nature and are later excreted by cells. Depending on the cells from which they originate, exosomes can play a role in tumor suppression or tumor progression. Tumor-derived exosomes (TDEs) have their own unique phenotypic functions. Evidence points to TDEs as key players involved in tumor growth, tumorigenesis, angiogenesis, dysregulation of immune cells and immune escape, metastasis, and resistance to therapies, as well as in promoting anti-tumor response. General exosomes, TDEs, and their influence on the TME are an area of promising research that may provide potential biomarkers for therapy, potentiation of anti-tumor response, development of exosome-based vaccines, and exosome-derived nanocarriers for drugs.

## INTRODUCTION

Amid the complexities of cancer in its development, growth, and metastasis, the tumor microenvironment (TME) is a unique platform of cancer biology that controls the local cellular environment in which a tumor exists. The TME is currently a very significant field of research, as increasing evidence points to the TME as pivotal for either promoting immune tumor rejection or protecting the tumor [1]. Thus, the TME may provide stimulatory factors that enhance tumor response and rejection. In contrast, the TME can also provide tumor cells with resistance mechanisms against therapeutic chemo and immune interventions, such as reducing drug penetration, conferring antiapoptotic advantages to cytotoxic immune cells, and withstanding genetic mutations [2-4]. Comprised of various cellular and non-cellular factions, the TME includes surrounding blood vessels, the extracellular matrix (ECM) and myofibroblasts, a variety of immune and regulatory cells, and a mix of signaling factors and cytokines directed against both immune cells and non-immune cells [2].

As one of the main components of the TME, tumor vasculature has been described as a defining characteristic of regulating cell growth and metastasis. In fact, sustained angiogenesis is considered one of the hallmarks of cancer, a term coined by Hanahan and Weinberg in their seminal article outlining the six key traits of cancer [5]. In normal tissue, the vasculature provides oxygen and nutrients required for cell growth, obligating nearly all cells to reside within 100 micrometers of a capillary vessel [5]. In the TME, however, vasculature is poorly organized, promoting hypoxia and limited growth factor feeding [3]. The reduced capability of tumor vasculature to effectively deliver molecules to the stroma is one of the challenges in providing anti-tumor agents as targeted therapies [6, 7].

The interstitial stroma of the TME is another contributing factor in tumor growth and development. It has been demonstrated that the ECM of tumors includes tissue rich in myofibroblastic cells, which are capable of supporting tumor progression, promoting angiogenesis, and providing protection against drugs [8, 9]. Normal stromal fibroblasts are transformed into cancer-associated fibroblasts (CAFs) through interactions with nearby cancer cells. These CAFs exhibit a unique phenotype of increased expression of ECM components and inflammatory cytokines [10]. CAFs can also develop a myofibroblastic phenotype, with the expression of smooth muscle cell markers, such as α-smooth muscle actin, and the production of transforming growth factor β (TGF-β) [10, 11]. The migration stimulating factor (MSF) and focal adhesions have been shown to contribute to an activated TME and the differentiation of cancer-associated myofibroblasts [10, 12]. MSF, as a potent mitogenic factor, promotes the migration of fibroblasts and endothelial cells in the TME and metabolically regulates stromal fibroblasts towards a glycolytic and tumor-promoting metabolism [10].

Multiple types of cells and cellular infiltrates can be found dispersed throughout the TME, and immune cells are certainly the majority of these cells. Monocytes, macrophages, T cells, B cells, myeloid cells, and natural killer (NK) cells are all encountered in the TME and play complex roles in immune activation, immune suppression, surveillance, tumor growth and metastasis, depending on the factors at hand [13-15].

Exosomes, an emerging molecular contributor in cancer biology, modulate and affect the TME and the well-established components of the TME - tumor vasculature, the ECM and myofibroblasts, and an assortment of immune cells. Tumor-derived exosomes (TDEs), in particular, are extracellular vesicles released from tumor cells, unique in their functions and origins [16, 17]. Evidence points to TDEs as key players involved in tumor growth, tumorigenesis, angiogenesis, immune escape, metastasis, and resistance to therapies [16, 18]. The present review will focus on both non-cancerous exosomes and TDEs, and their influence on the TME as an area of promising research providing potential biomarkers for therapy, reversing immune resistance, developing exosome-based vaccines, and generating exosome-derived nanocarriers for drugs [15, 16, 19, 20].

### Exosomes

#### Properties

**Figure 1:**
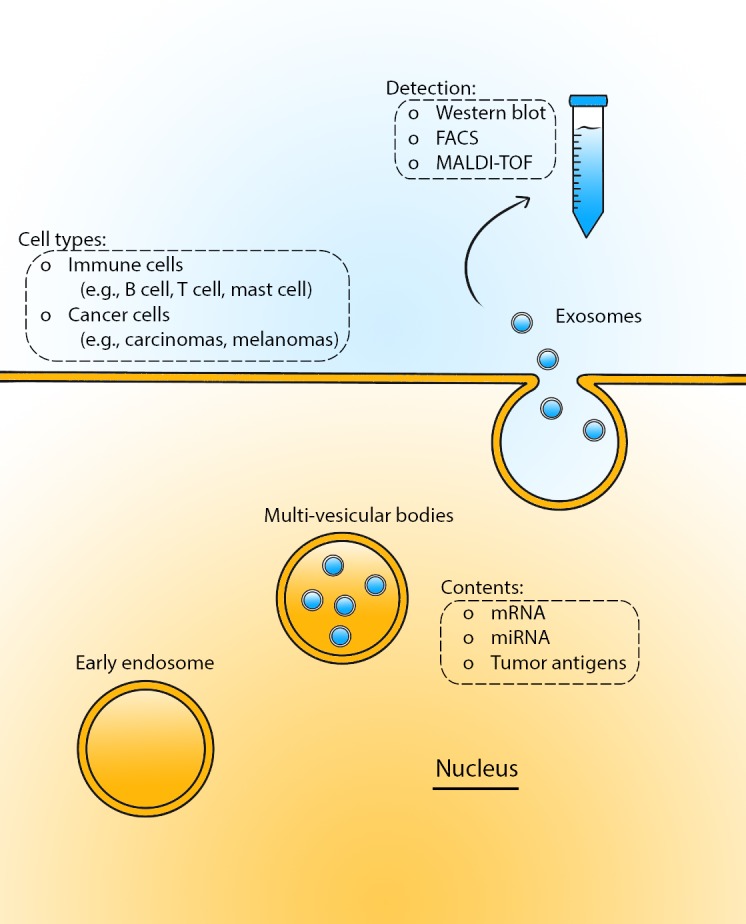
Exosome production and detection Exosomes are small (50–90 nm) membrane vesicles that are endocytic in origin and are released into the extracellular environment upon fusion with the plasma membrane. They are formed by multi-vesicular bodies, which release the exosomes into the surrounding environment. Isolation of exosomes is performed through differential centrifugation, from which the contents are then analyzed through Western Blog, FACS, and MALDI-TOF. Various cell types, including immune cells and cancer cells, release exosomes carrying a multitude of cargo, including but not limited to as microRNAs, mRNAs, and tumor antigens.

Exosomes are small (40-150 nm) membrane vesicles that are endocytic in origin and are released into the extracellular environment upon fusion with the plasma membrane [21-23]. Proteomic analysis of exosome profiles has uncovered their distinct entity and functions, which have helped to provide a theory of their biogenesis, namely, from multivesicular endosomes [24]. The physical properties of exosomes rely upon their purification from cell culture supernatants through differential centrifugation followed by sucrose density gradients [25]. Subsequently, further analyses are done through western blot, fluorescence-activated cell sorting (FACS), trypsin digestion, or peptide mapping by matrix-assisted laser desorption ionization-time of flight (MALDI-TOF) mass spectrometry (Figure 1) [26]. The exosomal protein profile is similar to that of molecules found in the endocytic pathway, plasma membrane, or cytosol. The cytosolic proteins that are involved in exosome biogenesis and function are mainly cytoskeleton-related (e.g. cofilin, profilin I) and signaling factors (e.g. rab 7, syntenin) [26]. The exosomal proteins are found on the cell surface and in endocytic vesicles, such as the CD9 cell surface protein in dendritic cells [26]. Despite having plasma membrane proteins, exosomes do not express cell surface proteins such as CD28 and CD45 (lymphocyte common antigen) in T-cell derived exosomes and types II/III Fc receptors in dendritic cell-derived exosomes [26, 27]. In addition, cytosolic proteins found in the exosomes are also present in the endocytic pathway, such as rab5/7 [28]. Exosomes are formed from inward budding of multivesicular endosomes that fuse with the plasma membrane resulting in the discharge of the exosomes into the extracellular environment [28-30]. The multivesicular endosomes are regulated by the endosomal sorting complexes required for the transport (ESCRT) pathway and the excretion of the exosomes controlled by Rab GTPases 27a and 27b [31-33].

#### Composition

The composition of exosomes can vary depending on the cell from which they originate. Many cells have the ability to release exosomes, including epithelial cells, tumor cells, and various immune cells, such as dendritic cells, B cells, T cells, and mast cells [21, 34]. Exosomes from mast cells have been shown to carry both mRNAs and microRNAs (miRNAs) that can be genetically exchanged between cells resulting in a specific protein production in the recipient cell [21]. In addition, it has been shown that the Major Histocompatibility Complex Class II molecules (MHC II) can be released by exosomes from mast cells and B cells [25, 35]. Tumor-derived exosomes contain tumor-associated antigens, which can be used to stimulate CD8 T cells for cross priming in immunotherapy [30]. The transfer of these contents is specific to ligands or signals on the recipient cells and exosomes. For example, exosomes from B lymphocytes express β1 and β2 integrins that adhere to fibroblasts [36]. Exosomes can bind to target cells through receptor-ligand interactions or by adhering to lipids on the cell membrane and can also be completely internalized by endocytosis or fused with the cell surface membrane [22, 37, 38].

#### Tumor-derived exosomes (TDEs)

**Figure 2:**
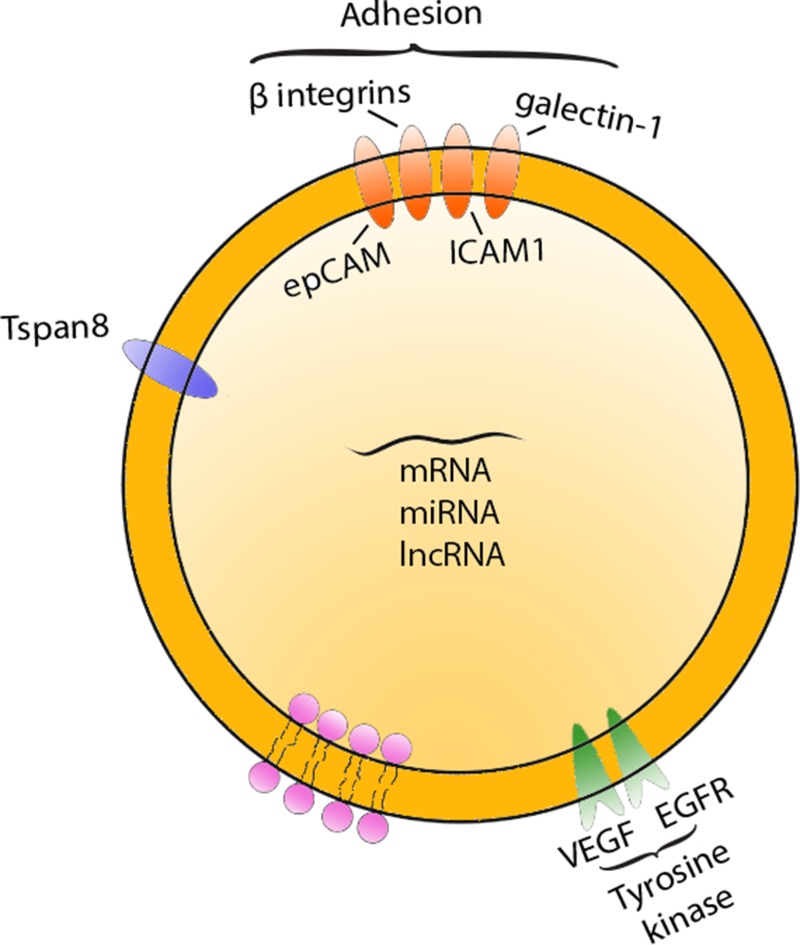
Tumor-derived exosome Tumor-derived exosomes (TDEs) are aid in the recruitment and reprogramming of the TME. They express various oncogenic factors that enhance tumorigenesis.

For cancer to metastasize, there has to be a constant communication between cancer cells and the distant host environment, and (TDEs) may contribute to this cross-talk by recruiting and reprogramming the TME [31]. Gliomas express the oncogenic epidermal growth factor receptor, known as EGFRvIII, that is exchanged between tumor cells by exosomes (Figure 2) [39]. Depending on the tumor cell origin, the contents of the TDEs vary. Exosomes from epithelial cancers contain the epithelial cell adhesion protein (EpCAM) and CD24, which are markers for poor prognosis in carcinomas [40]. Melanoma supernatants indicate the presence of exosomes containing melanoma antigen recognized by T cells 1 (Mart-1) and melanoma cell adhesion molecule (Mel-CAM) [30]. There is increasing evidence that exosomes can influence the TME by promoting angiogenesis and modulate the immune system in order to drive tumorigenesis (Table 1).

**Table 1:** Exosomal cargo affecting tumorigenesis

Exosome protein	Cell type	References
β1, β2 integrins	B lymphocytes	[34]
EGFRvIII	Gliomas	[38]
EpCAM	Carcinoma cells	[39]
CD24	Carcinoma cells	[39]
Mart-1	Melanoma cells	[28]
Mel-CAM	Melanoma cells	[28]
MHC class I A & B	Cervical cancer cells	[49]
ICAM-1	Prostate cancer cells	[52]
Galectin-1	Carcinoma cells	[64]
VEGF	Red blood cells	[69]
miR-214	Endothelial cells	[70]
lncRNA	Ovarian carcinoma cells	[73]
Tspan8	Adenocarcinoma cells	[74]
miR-21, 37e, 143	CAFs	[78]
Wnt10b	CAFs	[79]

Exosomes influence the TME by driving tumorigenesis.

#### Exosome-mediated immune regulation in the tumor microenvironment

In the early stages of cancer development, the process of immune surveillance eliminates precancerous and malignant cells through an immune-mediated response [41]. When this process is disrupted, however, then the immune system can no longer reject precancerous cells, and a tumor develops [42]. In the TME, tumor-derived exosomes are the mediators of intercellular communication, and as such, play a distinct role in the dysregulation of immune surveillance [13]. Considering the composition of TDEs - specifically RNAs, DNAs, and cytokines as vesicular contents - surrounding cells receive TDEs as signals to modulate their transcriptional and translational machineries [13, 21, 43]. To affect immune cells, TDEs can participate in both the direct and indirect antigen presentation to activate a CD8+ T cell response [38]. Direct presentation occurs as antigen-specific T cells directly engage an MHC-peptide complex on the TDE. On the other hand, indirect presentation involves the acquisition of antigen from the TDE by antigen presenting cells, like dendritic cells, which then process and present the antigen to CD8+ T cells [13]. The mechanism of activation of CD4+ T cells is less understood, although TDEs have been shown to directly activate primed, but not naïve, CD4+ T cells [44]. As such, TDEs carrying either native tumor antigen or an MHC-peptide complex have been shown to be key players in antigen presentation during immune surveillance [42, 45, 46].

**Figure 3:**
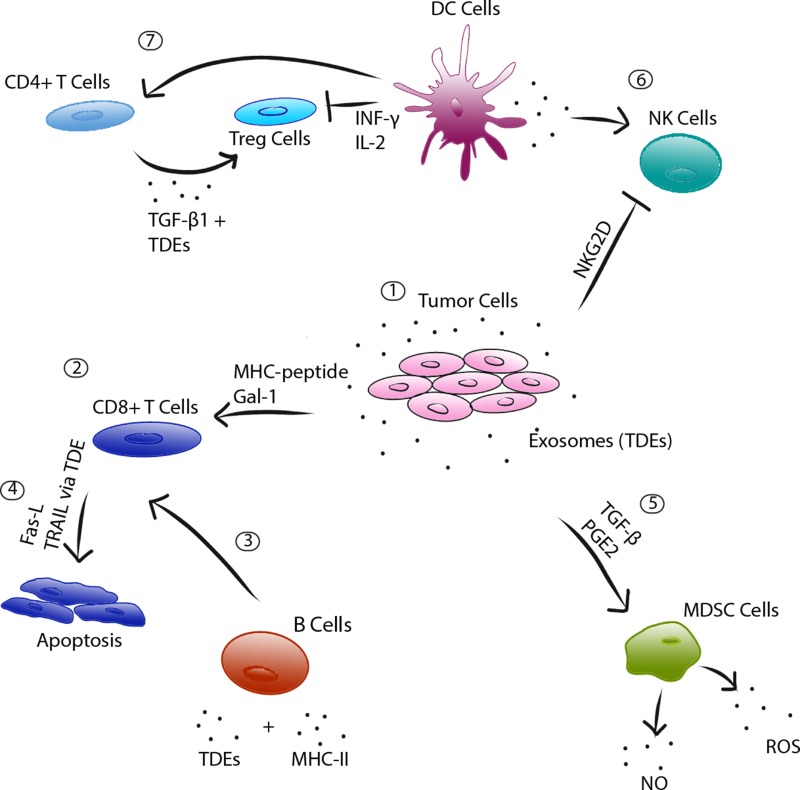
Exosome-mediated immune regulation 1) Tumor cells release exosomes (TDEs) that consequently affect numerous immune cells. 2) MHC-peptide complexes participate in direct antigen presentation to activate a CD8+ T cell response. 3) TDE antigens combined with MHC Class II molecules released from B cells also serve to activate CD8+ T cells. 4) However, TDEs can also induce apoptosis of activated CD8+ T cells, via TDE-bound FasL and exosomal TRAIL. 5) TDEs also drive the differentiation, via TGF-β and PGE_2_, of myeloid precursor cells to MDSCs, which release immunosuppressive factors NO and ROS. 6) Immune-cell derived exosomes, as from a dendritic cell, influence the TME and have been shown to boost NK cell immune response. However, TDEs combat this effect by downregulation of NKG2D receptors on NK cells. 7) DC-derived exosomes also stimulate CD4+ T cell response, which synergistically works with DC-derived exosomal cargo to inhibit Treg cells.

Through their intercellular signaling abilities and antigen presentation, exosomes are capable of both evoking an anti-tumor immune response and promoting tumor growth through inhibition of anti-tumor immunity [13]. The promotion of an immune response relies on pro-inflammatory mediators, which can be excreted in exosomes from macrophages, T cells, and dendritic cells [47]. Distinct from tumor-derived exosomes, these immune cell-derived exosomes activate the immune response primarily through the TNF-α pathway, which may then induce epithelial cells to secrete other pro-inflammatory cytokines, such as IL-8, RANTES, and additional TNF-α [48]. Exosomes released from dendritic cells may also induce other immune cells, such as NK cells, in an anti-tumor response (Figure 3) [49].

On the other hand, the immune response is largely mediated through TDEs in tumor evasion and in the impairment of the function and regulation of immune cells, thus promoting a tumorigenic environment. Firstly, an evasion of the immune response requires TDEs to prevent tumor antigen recognition by immune cells. One way in which this occurs is through the release of MHC class I related chain (MIC) ligands A and B, which bind to NKG2D receptors on NK cells and prevent tumor recognition while simultaneously downregulating NKG2D receptor expression on NK cells and T cells [50, 51]. Another process involves minimizing the recruitment of adaptive immune cells, which can occur when a TDE-bound form of intercellular adhesion molecule -1 (ICAM-1) interferes with leukocytic binding to vascular endothelial cells [52, 53]. Typically, ICAM-1 mediates leukocytic-endothelial binding to allow leukocytes to transmigrate into the tissue, but the exosomal form of ICAM-1 exhibits potent anti-binding properties [53]. Alternatively, TDEs can also induce decreased expression of CD3-ζ - a crucial T cell co-receptor - leading to a loss of T cell activation [54].

The second major mechanism through which TDEs inhibit the immune response lies in the dysregulation and dysfunction of immune cells. TDEs have been shown to interfere with monocyte differentiation into dendritic cells and directly interfere with dendritic cells’ bioactivity [55]. Myeloid-derived suppressor cells (MDSCs) are formed from myeloid precursor cells, driven into differentiation *via* TDE cargo of TGF-β and PGE_2_ [56, 57]. MDSCs then influence the immune response by producing immunosuppressive factors, such as nitric oxide and reactive oxidative species, and a secondary lack of DC cells needed for antigen presentation [58]. NK cells are another immune cell that TDEs may inhibit, with a decrease in their number and activity in the TME [59]. As previously mentioned, this is in part due to the binding and downregulation of the NKG2D receptor [51, 60]. In T cells, TDEs can both impair the activation of CD8+ T cells and induce apoptosis of activated T cells. Interference with TCR- and IL-2R signaling impairs T cell activation [59], while TDE-bound FasL and exosomal TRAIL can selectively induce apoptosis in T cells [61-64]. CD8+ T cells have also been shown to exhibit a suppressor phenotype, characterized by a loss of CD27/CD28 expression, mediated by galectin-1 found in TDEs [65]. The conversion of CD4+ T cells into Treg cells has also been shown to be mediated by TDEs through a TGF-β1 pathway (Figure 3) [66].

#### Exosomes crosstalk in the epithelium and the subepithelium in the TME

Tumor-derived exosomes release contents and communicate with other cells, such as fibroblast cells that function in the subepithelium and endothelial cells that line the interior of blood vessels. Angiogenesis, which is required for invasive tumor growth and metastasis, is dependent upon the sprouting of endothelial cells [67, 68]. Vascular endothelial growth factor (VEGF) and its receptors VEGFRs are key factors that drive vasculature, with VEGFR2 being the main regulator of the angiogenic effect of VEGF [69, 70]. When exosomes containing the miRNA 16/322/497/17 are co-cultured with endothelial cells, there is a decrease in the expression of VEGFR2 and subsequent inhibition of angiogenesis [70]. Conversely, when miR-214, which is found in tumor exosomes, is secreted by endothelial cells, it suppresses senescence and induces angiogenesis in endothelial cells [71].

Evidently, the exosomes can play a dual role, as they may participate in tumor suppression or promotion [72, 73]. But evidence suggests that tumor-derived exosomes promote angiogenesis in tumors. The epithelial ovarian cancer derived-exosomes can restore the migration of endothelial cells *via* the transfer of long non-coding RNAs (lncRNAs), which are RNA transcripts that are more than 200 nucleotides long but have been shown to be implicated in biological processes, such as gene expression [74]. Adenocarcinoma derived-exosomes containing Tspan8, which is a tetraspanin, induced angiogenesis by promoting VEGF-independent regulator of angiogenesis-related genes, such as chemokines CXCL5 and von Willebrand factor through the internalization of exosomes by endothelial cells [75]. Colorectal cancer-derived exosomes promote migration of endothelial cells by activating early growth response-1 (Egr-1) *via* the ERK1/2 and JNK signaling pathways [76].

Another cell involved in angiogenesis is the fibroblast, which produces growth factors, extracellular matrix, and chemokines that recruit endothelial cells [77]. The fibroblasts found in the tumor stroma are referred to as cancer-associated fibroblasts (CAFs) although their role in tumor progression is still unclear [78]. In breast cancer-derived exosomes, the exosomes in CAFs carry miRNAs -21, -37e, and -143, which are then released and promote a more aggressive phenotype of breast cancer by stimulating stemness and the epithelial mesenchymal transition (EMT), which is the loss of polarity and adhesion of epithelial cells into mesenchymal stem cells [79]. In breast cancer, p85*α*, which is a tumor suppressor, is downregulated [80]. The loss of p85α results in the transformation of fibroblasts in CAFs, which release exosomes containing Wnt10b that promote EMT induced by the canonical Wnt pathway [80]. In addition to release of content, the absence of content in CAF exosomes contributes to tumorigenesis. CAF-derived exosomes in hepatocellular carcinoma have reduced miR-320a, which could function as an antitumor microRNA [81]. In turn, overexpression of miR-320a in CAFs showed inhibition of tumorigenesis [81].

#### Exosomes-mediated anti-tumor therapies and suppression

Exosomes have been emerging to be attractive immune modulators in fighting cancer. Exosomes derived from B-cells and dendritic cells have shown the ability to induce antigen-specific T- and B- cell responses [82, 83]. Animal studies of dendritic cell (DC)-derived exosomes showed improved tumor microenvironment by a significant increase in CD8+ T lymphocytes, elevated levels of IFN-γ and interleukin-2, and decrease in CD25+Foxp3+ regulatory T (Treg) cells and of interleukin-10 and TGF-β in tumor sites [84]. Clinical trials of dendritic cell-derived exosomes showed antitumor immunity in patients with advanced non-small cell lung cancer (NSCLC) through boosting NKp30-dependent NK cell functions [85]. Exosomes derived from NK cells themselves have also been shown to have important therapeutic effects. Both *in vivo* and *in vitro* studies demonstrated that NK cell-derived exosomes exert cytotoxic effects on melanoma cells by presenting perforin and FasL, both of which are involved in apoptosis [86]. However, lymphocyte-derived exosomes are not the only immune modulators in the TME as TDEs are another source of vaccines for cancer immunotherapy. TDEs can promote immune function and inhibit tumor growth [87]. TDEs from lymphocytic leukemia cells significantly decrease TGF-β1 expression of DC cells; in addition, DC cells pulsed with those tumor exosomes can more effectively stimulate CD4+ T cell proliferation *in vitro* and Th1 cytokine secretion and induced a tumor-specific CTL response [87]. On the other hand, TDEs can also play a role in promoting tumor growth by immunosuppression. Pancreatic cancer-derived exosomes can decrease HLA-DR expression on CD14+ monocytes through the production of reactive oxidative stress (ROS) [88].

Recent evidence has demonstrated how exosomes are important mediators of intracellular communication in the TME and essential for cancer progression and metastasis [89, 90]. Cancer cells can produce exosomes full of tumor-promoting signaling molecules, such as Src tyrosine kinase, insulin-like growth factor 1 receptor (IGF-IR), and focal adhesion kinase (FAK) [91] Src, which signals through FAK, has been associated with many aspects of tumor progression, such as cell proliferation, metastasis, and angiogenesis; in addition, Src signaling is known to crosstalk with IGF-IR, which also promotes angiogenesis [91]. TDEs promote angiogenesis through the transfer of long intergenic non-coding RNA CCAT2 (linc-CCAT2) from glioma cells to endothelial cells [92].

RNA molecules are being shown to be common cargo in exosomes. MDSCs with miR-126a exosomes increased the expression of the inflammatory cytokine IL-13 that leads to increased blood vessel formation and promotes lung metastasis of breast cancer [93]. Small interfering RNAs (siRNAs), which have shown strong potential as therapeutic agents but difficult to deliver, are more permeable when loaded in exosomes for drug delivery [94].

As an exosomal cargo includes numerous proteins, lipids, and nucleic acids, valuable information can be garnered from their contents about their origins and function. In part, an effort has been made to characterize exosomes, and specifically TDEs, for use as biomarkers in disease diagnostics and therapeutics, or “theranostics” [54, 95, 96]. In this application, TDEs are collected and isolated from a liquid biopsy, which may include urine or blood serum [97] [98], and their contents are then purified and analyzed. Currently, the gold standard for exosomal purification includes differential centrifugation [96], although exosome isolation is still an active area of research [97, 99]. Biomarkers can be identified and differentiated as either up- or down-regulated through Western blot analysis for proteins or RT-qPCR for nucleic acids [97]. In particular, miRNAs in TDEs have proven useful as a potential source of biomarkers, for their stability, high specificity, and ease of collection [100]. Profile comparisons of miRNAs between tumor tissue and TDEs have shown to be highly homologous and can differentiate between benign pancreatic tumors and pancreatic carcinomas [101]. A few advantages of using TDEs include the prevention of invasive procedures to monitor disease and the ability to stratify patients into specific categories for therapy. Exosomal theranostics have already proven useful in detecting tumors of the central nervous system [98], in indicating nephrologies through urine collection and analysis [97], and in placental exosomal detection of preeclampsia [102]. However, although specific in distinguishing among diseases, miRNA analysis of TDEs cannot illustrate the severity of a disease [100]. Its use as a theranostic tool will continue to develop in the field of TDEs and in cancer biology as isolation, purification, and analytical techniques improve. It is projected that exosomes will not only prove useful as biomarkers for early disease detection, but they may also serve as biomarkers to predict a patient's response to drugs [103]. Moreover, fluctuating concentrations of exosomes circulating in the serum, bile, or other organ systems are expected to be useful as biomarkers indicating progression of a disease [104].

## CONCLUDING REMARKS AND PERSPECTIVES

Exosomes are small membrane vesicles that are formed from inward budding of multivesicular endosomes and that then fuse with the plasma membrane resulting in the discharge of the exosomes into the extracellular environment [28-30]. While immunotherapy serves to target TDEs, other modalities of treatment seek to utilize TDEs for their advantageous properties, such as their size, composition, and tumor-targeting capacity [105]. Several current emerging therapies utilizing exosomes include anti-tumor vaccines, delivery vehicles for drugs, and gene therapy. First, TDEs offer a unique approach to develop anti-tumor vaccines due to their integrated function in tumor cell intercommunication. It has been shown that TDEs derived from pancreatic cancer cell lines were able to effectively decrease tumor cell proliferation though downregulation of the Notch-1 signaling pathway and activation of a mitochondria-dependent apoptosis mechanism [106]. These TDEs, rich in lipid rafts, served as signals to decrease the expression of Hes-1, the intranuclear target of Notch-1, which led to apoptosis after a cell cycle arrest in the G_0_G_1_ phase [106]. Besides TDEs, other types of exosomes, such as dendritic cell-derived exosomes, could be utilized to target tumors by priming them with tumor-associated antigens and thus eliciting an NK cell response [107, 108]. The small size and structural makeup of exosomes also make them useful in serving as potential nanocarriers for drug delivery. They can be packaged with small molecules, proteins, or nucleic acids and administered to patients, avoiding first pass metabolism of the liver and absorption in the gut, with the ability to cross difficult membranes such as the blood brain barrier [109]. Cytotoxic drugs can also be effectively delivered to target cells without altering their mechanism of action, as illustrated in the delivery of acridine orange to melanoma cells [110]. However, it remains a challenge to be able to properly design exosomes for drug delivery and to avoid an unwanted immune reaction [109, 111]. The delivery of miRNAs or RNAis *via* exosomes allows for rapid gene expression in target cells while avoiding the lysosomal pathway through a direct cytosolic delivery [111-113]. In addition, there is evidence of transport and excretion by proteins and lipids by exosomes. Exosomes may transport lipids such as sphingolipids, cholesterol, and ceramide [114, 115]. Silverman et. al. identified exosome-based secretion as the main mechanism for protein secretion by Leishmania and showed that exosomes deliver proteins to host target cells [116]. Many proteins are found on exosomes that include membrane transport proteins, tetraspannins, heat shock proteins, and multi-vesicular proteins [103, 117].

The biogenesis of exosomes is carried out mainly by 2 types of pathways: endosomal sorting complex required for transport (ESCRT)-dependent and ESCRT independent. The-ESCRT dependent pathway relays on a complex of various proteins as well as certain carbohydrate molecules for the successful biogenesis of exosomes through multi vesicular binding (MVB) formation. ESCRT consists of a five distinct protein complex, namely, ESCRTs 0, I, II, and III, AAA ATPase and Vps4. This process is initiated in the endosomal system [118]. A report by Trajkovic et al. (2008) examined the ESCRT-independent pathway of exosome generation and reported that Ceramide has a significant role in MVB formation [119]. Further, 5 Rab GTPases were reported in the exosome biogenesis from HeLa cells [118, 120]. Several proteins were involved in the ESCRT-independent pathway, including sphingomylinase-2 [121], sphingosine-1 phosphate receptors [122], transferrin receptors [123], and p53 [124].

A better understanding of exosomes and their role in cancer have led to the development of exosomal theranostics in order to use exosomes for both diagnostic and therapeutic purposes. Exosomes have been gaining notice for their function in the TME, and TDEs, in particular, provide a promising direction for cancer treatment as mechanisms for better drug delivery, tumor suppression, and immunoregulation due to their advantageous size, composition, and homing capabilities (Table 2). Yet the manifold exosomal functions present another face of exosomes as in their role in cancer progression. The development of cancer involves the complex interaction of cells and signaling molecules in the TME and exosomes have been shown to promote tumor growth though the inhibition of anti-tumor immunity and the development of angiogenesis. Future research would fully take advantage of exosomes’ ubiquitous presence in eukaryotic cells as they appear to provide a plethora of conduits for anti-cancer therapy.

**Table 2:** Exosome-based applications and therapies in cancer

Immune Cell Derived Exosomes Activities	Pros	Cons
**DC-derived**	Improved TME by increased CD8+ T cells, IFN-γ, and IL-2 Decreased Treg cells, IL-10, and TGF-β in tumor sites	Challenges in vesicle engineering and delivery
**NK-derived**	Perforin and FasL lead to apoptosis in melanoma	Challenges in vesicle engineering and delivery
**MDSC-derived**	Potential target of anti-tumor therapies	miR-126a exosomes lead to increased IL-13 expression and angiogenesis in tumors Challenges in vesicle engineering and delivery
**TDEs**	Decreased tumor proliferation via NOTCH1 pathway	Contains tumor-promoting signals like IGF-1R, FAK, and Src Promotes angiogenesis (linc CCAT2)
**Nanocarriers**	Cross difficult membranes (Blood brain barrier) Delivery of gene therapy	Proper design is a challenge Need to avoid immune response
**Diagnostics**	Highly homologous mRNA offers stability and specificity during collection	Cannot distinguish disease severity Difficult isolation of other exosomal cargo

Advantages and disadvantages of exosome-based therapies, including immune cell derived exosomes and TDEs, and exosomal applications as nanocarriers and in diagnostics are listed.

## OVERALL

Due to the complexity of exosomes and their contrasting roles in cancer, several areas need attention, including (1) better methods for isolating cancer exosomes (2) better understanding how the cargos in exosomes are packed particularly that cancer cells are heterogeneous and in each cell the nature of the cargo is different; this will facilitate early diagnosis and response to treatments using exosomes (3) undertaking clinical trials in different types of cancers in order to validate the use of exosomes as diagnostics and therapeutics (4) the identification of biomarkers of exosomes in response to various therapies. (5) the use of exosomes as delivery vehicles for drugs, antigens, nucleic acids, etc., and (6) establishment of GMP facilities for the manufacture, storage, and stability for the administration of therapeutic exosomes of high quality and with high safety.

## Abbreviations

CAF: cancer-associated fibroblast
CD8: cluster of differentiation 8
CD9: cluster of differentiation 9
CD24: cluster of differentiation 24
CD28: cluster of differentiation 28
CD45: cluster of differentiation 45
DC cell: dendritic cell
EpCAM: epithelial cell adhesion protein
ECM: extracellular matrix
EGFRvIII: epidermal growth factor receptor
Egr-1: early growth response-1
EMT: epithelial mesenchymal transition
ESCRT: endosomal sorting complexes required for transport
FACS: fluorescence-activated cell sorting
FAK: focal adhesion kinase
ICAM-1: intercellular adhesion molecule -1
IGF-IR: insulin-like growth factor 1 receptor
lncRNA: long non-coding RNA
Mart-1: melanoma antigen recognized by T cells 1
MALDI-TOF: matrix-assisted laser desorption ionization-time of flight
Mel-CAM: melanoma cell adhesion molecule
MDSC: myeloid derived suppressor cell
MHC II: Major Histocompatibility Complex Class II
MSF: migration stimulating factor
miRNA: microRNA
NK cells: natural killer cells
NSCLC: non-small cell lung cancer
PGE2: prostaglandin E2
RANTES: regulated on activation, normal T cell expressed and secreted
siRNA: small-interfering RNA
TDE: tumor-derived exosome
TGF- β: transforming growth factor β
TME: tumor microenvironment
Treg: regulatory T cell
VEGFR: Vascular endothelial growth factor
